# Framingham Heart Study 100K Project: genome-wide associations for blood pressure and arterial stiffness

**DOI:** 10.1186/1471-2350-8-S1-S3

**Published:** 2007-09-19

**Authors:** Daniel Levy, Martin G Larson, Emelia J Benjamin, Christopher Newton-Cheh, Thomas J Wang, Shih-Jen Hwang, Ramachandran S Vasan, Gary F Mitchell

**Affiliations:** 1 The National Heart, Lung, and Blood Institute's Framingham Heart Study, Framingham, MA, USA; 2 The National Heart, Lung, and Blood Institute, Bethesda, MD, USA; 3 Boston University School of Medicine, Boston MA, USA; 4 Department of Mathematics, Boston University, Boston, MA, USA; 5 Division of Cardiology, Boston Medical Center, Boston, MA, USA; 6 Division of Cardiology, Massachusetts General Hospital and Harvard Medical School, Boston, MA, USA; 7 Cardiovascular Engineering Inc., Waltham, MA, USA

## Abstract

**Background:**

About one quarter of adults are hypertensive and high blood pressure carries increased risk for heart disease, stroke, kidney disease and death. Increased arterial stiffness is a key factor in the pathogenesis of systolic hypertension and cardiovascular disease. Substantial heritability of blood-pressure (BP) and arterial-stiffness suggests important genetic contributions.

**Methods:**

In Framingham Heart Study families, we analyzed genome-wide SNP (Affymetrix 100K GeneChip) associations with systolic (SBP) and diastolic (DBP) BP at a single examination in 1971–1975 (n = 1260), at a recent examination in 1998–2001 (n = 1233), and long-term averaged SBP and DBP from 1971–2001 (n = 1327, mean age 52 years, 54% women) and with arterial stiffness measured by arterial tonometry (carotid-femoral and carotid-brachial pulse wave velocity, forward and reflected pressure wave amplitude, and mean arterial pressure; 1998–2001, n = 644). In primary analyses we used generalized estimating equations in models for an additive genetic effect to test associations between SNPs and phenotypes of interest using multivariable-adjusted residuals. A total of 70,987 autosomal SNPs with minor allele frequency ≥ 0.10, genotype call rate ≥ 0.80, and Hardy-Weinberg equilibrium p ≥ 0.001 were analyzed. We also tested for association of 69 SNPs in six renin-angiotensin-aldosterone pathway genes with BP and arterial stiffness phenotypes as part of a candidate gene search.

**Results:**

In the primary analyses, none of the associations attained genome-wide significance. For the six BP phenotypes, seven SNPs yielded p values < 10^-5^. The lowest p-values for SBP and DBP respectively were rs10493340 (p = 1.7 × 10^-6^) and rs1963982 (p = 3.3 × 10^-6^). For the five tonometry phenotypes, five SNPs had p values < 10^-5^; lowest p-values were for reflected wave (rs6063312, p = 2.1 × 10^-6^) and carotid-brachial pulse wave velocity (rs770189, p = 2.5 × 10^-6^) in MEF2C, a regulator of cardiac morphogenesis. We found only weak association of SNPs in the renin-angiotensin-aldosterone pathway with BP or arterial stiffness.

**Conclusion:**

These results of genome-wide association testing for blood pressure and arterial stiffness phenotypes in an unselected community-based sample of adults may aid in the identification of the genetic basis of hypertension and arterial disease, help identify high risk individuals, and guide novel therapies for hypertension. Additional studies are needed to replicate any associations identified in these analyses.

## Background

Hypertension affects about one quarter of adults in industrialized countries [1] and carries a substantial burden of risk for cardiovascular disease (CVD), kidney disease, and death [2]. Increased arterial stiffness is a key factor in the pathogenesis of hypertension in older people and it contributes to the development of hypertensive target organ damage, CVD, and death [3-5]. Substantial heritability of blood pressure [6] and arterial stiffness [7]), as measured by arterial tonometry, points to genetic contributions to these cardiovascular phenotypes.

The search for genetic variants contributing to hypertension and arterial stiffness has focused on complementary approaches: linkage applied to rare Mendelian blood pressure disorders and to large family-based studies to identify positional candidate genes, and the study of biologically plausible candidate genes selected by virtue of their role in blood pressure regulation or vascular properties. A great deal is known about mutations responsible for Mendelian blood pressure disorders [8], but neither these rare variants nor more common variants in these genes account for substantial blood pressure variation in the general population. Similarly, although numerous linkage [9] and candidate gene association studies [10] have been conducted, there is a paucity of evidence that common genetic variation contributes to alterations in blood pressure or arterial stiffness in the general population.

Genome-wide association offers the opportunity to conduct analysis of common genetic variants unconstrained by prior knowledge of biological pathways in relation to phenotypes of interest. This approach succeeded in identifying the association of complement factor H with age-related macular degeneration [11]. The Framingham Heart Study, which enrolled participants without regard to phenotype status, provides a setting for a genome-wide association study in a community-based sample in which selection bias is inherently low. In addition, because of the familial structure of the study, it also provides an opportunity to use genome-wide SNP data for family based association testing (FBAT) and linkage analyses.

In this report we provide results of a genome-wide association study of blood pressure and arterial stiffness, including results of generalized estimating equation (GEE) association testing, FBAT, and linkage, as well as a summary of associations of these phenotypes with candidate genes in the renin-angiotensin-aldosterone pathways.

## Methods

### Study sample

The Framingham Heart Study began in 1948 when 5209 men and women from Framingham, Mass, who were between 28 and 62 years of age were recruited to participate in an observational study [12]. Subjects underwent a medical history, physician-administered physical examination including blood pressure measurement, laboratory tests, and electrocardiography. Examinations have been repeated every 2 years. In 1971, 5124 offspring and spouses of offspring of original participants were recruited into the Framingham Offspring Cohort [13]. The offspring cohort was reexamined approximately every 4 years, except for an 8 year interval between their initial and second visit. All subjects gave written informed consent before each clinic visit, and the examination protocol was approved by the Institutional Review Board at Boston Medical Center (Boston, Mass).

### Blood pressure phenotypes

At each clinic visit, the examining physician measured the systolic and diastolic BP in the left arm using a mercury column sphygmomanometer. BP was measured twice at each visit, with the exception of the first Offspring Cohort clinic visit, when it was measured once in about half the participants. Systolic and diastolic pressures were determined by the first and fifth Korotkoff sounds, respectively, and the two BP measurements were averaged to derive the systolic and diastolic pressures for that examination.

Examination cycles for the two cohorts were overlaid temporally as follows [offspring cohort/original cohort (earliest - latest year)]: examination 1/examination 12 (1971–1975), examination 2/examination 16 (1979–1983), examination 3/examination 18 (1983–1987), examination 4/examination 20 (1986–1991), examination 5/examination 22 (1990–1995), examination 6/examination 24 (1995–1998) and examination 7/examination 26 (1998–2001). Referring to offspring cycle numbers, the six BP phenotypes analyzed for this investigation were residuals for SBP and DBP at Examination 1, at Examination 7, and average of residuals from available Examinations 1 to 7. BP was imputed for treated observations as previously described [6]. No adjustment was made for untreated observations, which constituted the vast majority of BP values. Systolic and diastolic BP phenotypes were analyzed independently. Residuals were obtained from cohort- and examination-specific regression models accounting for sex, age and BMI; for DBP, age-squared was added. For inclusion in long-term BP analyses, each participant had to have BP measured on at least three examinations over a period of 12 years or more.

### Arterial stiffness phenotypes

Arterial tonometry for assessment of arterial stiffness was conducted on Offspring Cohort participants attending their 7th clinic examination. Five primary tonometry phenotypes were analyzed: carotid-femoral and carotid-brachial pulse wave velocity, forward and reflected pressure wave amplitude, and mean arterial pressure. Tonometry was performed in the supine position after 5 minutes of rest. Arterial tonometry with simultaneous ECG recording was obtained from brachial, radial, femoral and carotid arteries using a commercially available tonometer (SPT-301, Millar Instruments, Houston, TX). Carotid-brachial, carotid-radial and carotid-femoral PWV were calculated as previously described [14]. Mean arterial pressure was calculated from the planimetered brachial arterial tracing after calibration to the brachial blood pressure, which was obtained by an oscillometric device. Forward pressure wave amplitude was defined as the difference between pressure at the waveform foot and pressure at the first systolic inflection point or peak of the carotid pressure waveform; reflected pressure wave amplitude was defined as the difference between the central systolic pressure and the pressure at the forward wave peak. Sex-specific regressions were conducted for each tonometry phenotype with the following covariates: age, age^2^, height, weight, to generate sex-specific residuals.

### Genotyping methods

Details of the genotyping methods are available in the Executive Summary [15]. Briefly, 112990 autosomal SNPs on the Affymetrix 100K chip were genotyped in the Boston University School of Medicine Genetics Laboratory on the Framingham Heart Study family plate set. SNPs were excluded for the following reasons: minor allele frequency <10% (n = 38062); call rate <80% (n = 2346); Hardy Weinberg equilibrium p value < 0.001 (n = 1595), leaving 70,987 SNPs available for analysis.

### Statistical methods

Standardized multivariable-adjusted blood pressure and tonometry residuals were generated as described above. Table 1 lists the covariates used for each phenotype. As described in the Executive Summary [15], we conducted association testing using family based association testing (FBAT), and generalized estimating equations (GEE) applied to the additive genetic effects model. In secondary analyses that used the GEE general genetic effects model, which is more sensitive to recessive genetic effects, to be more conservative, we limited analyses to two phenotypes: long-term SBP and long-term DBP, and we limited eligible SNPs to those with a minor allele frequency >= 0.20 and Hardy-Weinberg equilibrium p value >= 0.05. The software package Merlin [16] was used to compute exact identity by descent linkage probabilities for allele sharing, and linkage analysis by variance component method was carried out SOLAR using 11,200 SNPs and STRs. Heritability was estimated using variance-components methods (SOLAR). For BP, 2155 study participants were used for examination 1 SBP and DBP, 1479 for examination 7, and 2009 for long-term average; 770 individuals were used in heritability analysis of arterial stiffness phenotypes.

**Table 1:** Phenotype List

		Exam cycle/s			
	N*	Offspring	Cohort	Adjustment	Covariates

**Primary Phenotypes**					

Blood Pressure					

SBP 1	2	1	12	Age and sex, multivariable	Cohort, sex, age, BMI
SBP 7	2	7	26	Age and sex, multivariable	Cohort, sex, age, BMI
SBP 1–7	2	Mean of exams 1–7	Mean of exams 12, 16, 18, 20, 22, 24, 26	Age and sex, multivariable	Cohort, sex, age, BMI
DBP 1	2	1	12	Age and sex, multivariable	Cohort, sex, age, BMI
DBP 7	2	7	26	Age and sex, multivariable	Cohort, sex, age, BMI
DBP 1–7	2	Mean of exams 1–7	Mean of exams 12, 16, 18, 20, 22, 24, 26	Age and sex, multivariable	Cohort, sex, age, BMI

Tonometry					

Carotid-femoral PWV	2	7	Not included	Age and sex, multivariable	Sex, age, age^2, height, weight
Carotid-brachial PWV	2	7	Not included	Age and sex, multivariable	Sex, age, age^2, height, weight
Forward pressure wave	2	7	Not included	Age and sex, multivariable	Sex, age, age^2, height, weight
Reflected pressure wave	2	7	Not included	Age and sex, multivariable	Sex, age, age^2, height, weight
Mean arterial pressure	2	7	Not included	Age and sex, multivariable	Sex, age, age^2, height, weight

**Secondary Phenotypes^**					

Blood Pressure					

DBP 2	2	2	16	Age and sex, multivariable	Cohort, sex, age, BMI
DBP 3	2	3	18	Age and sex, multivariable	Cohort, sex, age, BMI
DBP 4	2	4	20	Age and sex, multivariable	Cohort, sex, age, BMI
DBP 5	2	5	22	Age and sex, multivariable	Cohort, sex, age, BMI
DBP 6	2	6	24	Age and sex, multivariable	Cohort, sex, age, BMI
SBP 2	2	2	16	Age and sex, multivariable	Cohort, sex, age, BMI
SBP 3	2	3	18	Age and sex, multivariable	Cohort, sex, age, BMI
SBP4	2	4	20	Age and sex, multivariable	Cohort, sex, age, BMI
SBP 5	2	5	22	Age and sex, multivariable	Cohort, sex, age, BMI
SBP 6	2	6	24	Age and sex, multivariable	Cohort, sex, age, BMI
PP 1	2	1	12	Age and sex, multivariable	Cohort, sex, age, BMI
PP 2	2	2	16	Age and sex, multivariable	Cohort, sex, age, BMI
PP 3	2	3	18	Age and sex, multivariable	Cohort, sex, age, BMI
PP 4	2	4	20	Age and sex, multivariable	Cohort, sex, age, BMI
PP 5	2	5	22	Age and sex, multivariable	Cohort, sex, age, BMI
PP 6	2	6	24	Age and sex, multivariable	Cohort, sex, age, BMI
PP 7	2	7	26	Age and sex, multivariable	Cohort, sex, age, BMI
PP 1–7	2	Mean of exams 1–7	Mean of exams 12, 16, 18, 20, 22, 24, 26	Age and sex, multivariable	Cohort, sex, age, BMI

Tonometry					

1/CF-PWV	2	7	Not included	Age and sex, multivariable	Sex, age, age^2, height, weight
AI	2	7	Not included	Age and sex, multivariable	Sex, age, age^2, height, weight
CPP	2	7	Not included	Age and sex, multivariable	Sex, age, age^2, height, weight
CR-PWV	2	7	Not included	Age and sex, multivariable	Sex, age, age^2, height, weight
DBP-osc	2	7	Not included	Age and sex, multivariable	Sex, age, age^2, height, weight
PA-1	2	7	Not included	Age and sex, multivariable	Sex, age, age^2, height, weight
PA-2	2	7	Not included	Age and sex, multivariable	Sex, age, age^2, height, weight
PP-osc	2	7	Not included	Age and sex, multivariable	Sex, age, age^2, height, weight
RWTT	2	7	Not included	Age and sex, multivariable	Sex, age, age^2, height, weight
SBP-osc	2	7	Not included	Age and sex, multivariable	Sex, age, age^2, height, weight

*n = number of phenotypes analyzed

^Association results for primary and secondary phenotypes are available on the worldwide web at: http://web.ncbi.nlm.nih.gov/projects/gap/framingham/cgi-bin/study.cgi?id=phs000007

AI = augmentation index; CPP = central pulse pressure; CB-PWV = carotid brachial pulse wave velocity; CF-PWV = carotid-femoral pulse wave velocity; CR-PWV = carotid-radial pulse wave velocity; DBP = diastolic blood pressure; DBP-osc = brachial DBP by oscillometric device; FW = forward wave amplitude; MAP = mean arterial pressure; PA-1 = apparent peripheral amplification; PA-2 = true peripheral amplification; PP = pulse pressure; PP-osc = brachial PP by oscillometric device; RW = reflected wave amplitude; RWTT = reflected wave transit time; SBP = systolic blood pressure; SBP-osc = brachial SBP by oscillometric device; 1/CF-PWV = inverse of CF-PWV.

### Candidate gene analyses

GEE and FBAT additive genetic effect models were run for SNPs in or near 6 genes in the renin-angiotensin-aldosterone pathways. These genes were selected a priori because of a substantial body of literature implicating them in hypertension and altered vascular properties. All SNPs from 200 Kb proximal to the start and extending to 200 kb of the terminus of each gene were included in analysis providing the minor allele frequency was >= 0.1, the genotype call rate was 0.8, and the Hardy-Weinberg equilibrium p value was >= 0.001.

## Results

The six primary BP phenotypes were examination 1 SBP and DBP (n = 1260), examination 7 SBP and DBP (n = 1233), and long-term averaged SBP and DBP (n = 1327). The five primary arterial stiffness phenotypes were carotid-femoral and carotid-brachial pulse wave velocity, forward and reflected pressure wave amplitude, and mean arterial pressure (n = 644). The study sample available for BP phenotypes included up to 1327 individuals (mean age 52 years, 54% women for the long-term SBP and DBP phenotypes). The complete list of blood pressure and arterial stiffness phenotypes analyzed and the covariates used in generating sex-specific standardized residuals for each phenotype are listed in Table 1. Full disclosure of all GEE and FBAT associations for the traits listed in Table 1 can be found at the National Center for Biotechnology Information dbGaP website: http://web.ncbi.nlm.nih.gov/projects/gap/framingham/cgi-bin/study.cgi?id=phs000007.

Results of primary GEE models for an additive genetic effect for DBP, SBP, and arterial stiffness phenotypes are presented in Table 2a. None of the association results attained genome-wide significance. The lowest p values for DBP, SBP, and arterial stiffness phenotypes, respectively, were rs1963982 (p = 3.31 × 10^-6^), rs10493340 (p = 1.7 × 10^-6^), and rs6063312 (p = 2.1 × 10^-6 ^for reflected wave amplitude). For the same three phenotype groups the number of associations with p values < 10^-5 ^were 6, 1, and 5, respectively.

**Table 2:** Results of GEE and FBAT Additive Genetic Effects: Association, Linkage, and Heritability of Blood Pressure and Arterial Stiffness Phenotypes

2a. Results of GEE Additive Genetic Effects Models							
**Phenotype**	**Exam**	**SNP**	**Chr.**	**Position**	**GEE P value**	**FBAT P value**	**Gene**

**Diastolic Blood Pressure**							

DBP	7	rs1963982	8	73,269,470	**3.31 × 10^-6^**	0.002	
DBP	1	rs935334	14	75,683,431	**3.32 × 10^-6^**	0.002	
DBP	7	rs4370013	3	2,629,691	**3.73 × 10^-6^**	0.032	CNTN4
DBP	7	rs10491334	5	110,800,303	**4.47 × 10^-6^**	0.133	CAMK4
DBP	1	rs2121070	14	75,720,517	**4.88 × 10^-6^**	0.02	C14orf118
DBP	1	rs2509458	6	88,709,299	**6.94 × 10^-6^**	0.001	
DBP	7	rs6950982	7	100,360,038	**1.22 × 10^-5^**	0.036	TRIM56, SERPINE1, AP1S1
DBP	7	rs10510911	3	63,678,681	**1.65 × 10^-5^**	0.021	
DBP	1	rs1816088	5	39,897,583	**1.73 × 10^-5^**	0.012	
DBP	7	rs1519592	6	140,585,329	**1.89 × 10^-5^**	2.83 × 10^-4^	

**Systolic Blood Pressure**							

SBP	1	rs10493340	1	63,303,150	**1.69 × 10^-6^**	0.13	
SBP	7	rs1841055	4	70,039,785	**2.07 × 10^-5^**	0.003	UGT2A3
SBP	1	rs2035254	3	107,292,420	**2.20 × 10^-5^**	0.046	
SBP	1–7	rs1408263	6	18,515,722	**2.43 × 10^-5^**	0.121	IBRDC2
SBP	7	rs1408113	9	113,822,387	**2.54 × 10^-5^**	0.034	ZNF618
SBP	7	rs629448	9	26,263,322	**3.14 × 10^-5^**	0.011	
SBP	7	rs10485320	6	47,884,860	**3.28 × 10^-5^**	0.012	OPN5
SBP	7	rs10512889	5	6,921,922	**4.17 × 10^-5^**	0.008	
SBP	1	rs1328925	4	159,547,895	**4.32 × 10^-5^**	0.118	TMEM144
SBP	7	rs9321764	6	140,532,157	**4.39 × 10^-5^**	4.76 × 10^-4^	

**Tonometry Phenotypes**							

RW	7	rs6063312	20	46,776,466	**2.09 × 10^-6^**	0.063	PREX1
CB-PWV	7	rs770189	5	88,124,195	**2.53 × 10^-6^**	0.005	MEF2C
CB-PWV	7	rs10514688	3	34,937,673	**5.66 × 10^-6^**	0.027	
CB-PWV	7	rs7042864	9	107,951,862	**6.13 × 10^-6^**	0.077	
MAP	7	rs1322512	6	153,040,067	**7.76 × 10^-6^**	0.038	SYNE1
FW	7	rs348384	19	6,503,386	**1.16 × 10^-5^**	0.058	TUBB4, TNFSF9, TNFSF7
RW	7	rs10507514	13	42,132,814	**1.28 × 10^-5^**	0.066	TNFSF11
FW	7	rs3793427	8	17,188,201	**1.43 × 10^-5^**	0.059	VPS37A
RW	7	rs10506928	12	85,003,844	**1.62 × 10^-5^**	0.021	
FW	7	rs4075701	2	116,146,020	**1.63 × 10^-5^**	0.025	
RW	7	rs11784583	8	103,154,213	**3.83 × 10^-5^**	0.036	
RW	7	rs10513957	18	65,039,417	**4.15 × 10^-5^**	0.019	
CF-PWV	7	rs10506440	12	60,993,853	**4.18 × 10^-5^**	0.064	USP15
RW	7	rs1197850	13	34,828,744	**4.57 × 10^-5^**	0.042	

**2b. Results of FBAT Additive Genetic Effects Models**							

**Phenotype**	**Exam**	**SNP**	**Chr.**	**Position**	**GEE P value**	**FBAT P Value**	**Gene**

DBP	1	rs1590919	13	104,000,000	0.079	**1.42 × 10^-6^**	
DBP	1–7	rs636864	6	150,000,000	4.49 × 10^-4^	**1.55 × 10^-6^**	
DBP	1	rs726698	2	35,366,992	0.02	**1.15 × 10^-5^**	
DBP	7	rs1338657	6	103,000,000	0.001	**2.57 × 10^-5^**	
DBP	1–7	rs10506595	12	69,191,621	0.133	**3.40 × 10^-5^**	PTPRB
DBP	7	rs9311171	3	37,971,481	0.025	**4.03 × 10^-5^**	CTDSPL
DBP	1	rs10520569	15	82,520,393	0.577	**4.24 × 10^-5^**	ADAMTSL3
DBP	7	rs4514016	8	120,000,000	3.70 × 10^-5^	**4.52 × 10^-5^**	SAMD12
DBP	7	rs2322509	8	27,052,291	0.172	**4.91 × 10^-5^**	
DBP	1–7	rs10504389	8	66,718,741	0.1	**5.53 × 10^-5^**	ARMC1, MTFR1

SBP	1	rs1588260	5	121,000,000	0.001	**3.43 × 10^-6^**	
SBP	1	rs726698	2	35,366,992	0.023	**2.70 × 10^-5^**	
SBP	7	rs963328	1	209,000,000	0.036	**3.01 × 10^-5^**	
SBP	7	rs729053	18	50,960,679	0.008	**3.41 × 10^-5^**	
SBP	7	rs1434939	8	69,666,816	0.004	**4.97 × 10^-5^**	
SBP	1–7	rs10498500	14	62,030,261	0.005	**6.25 × 10^-5^**	
SBP	1	rs3853241	5	166,000,000	0.003	**6.25 × 10^-5^**	
SBP	1–7	rs1590919	13	104,000,000	0.14	**6.66 × 10^-5^**	
SBP	1	rs6763833	3	65,953,132	0.374	**8.18 × 10^-5^**	MAGI1
SBP	7	rs6940110	6	10,377,050	0.145	**8.42 × 10^-5^**	

FW	7	rs1539377	9	81,441,976	5.48 × 10^-5^	**5.26 × 10^-6^**	TLE1
RW	7	rs792833	3	101,000,000	0.123	**6.01 × 10^-6^**	COL8A1
MAP	7	rs10495191	1	219,000,000	0.007	**1.46 × 10^-5^**	TAF1A
CB-PWV	7	rs10494786	1	196,000,000	0.079	**1.56 × 10^-5^**	
CB-PWV	7	rs2160595	18	61,742,129	0.001	**2.38 × 10^-5^**	CDH7
FW	7	rs28899	5	82,798,839	0.001	**2.99 × 10^-5^**	VCAN
CF-PWV	7	rs1349721	4	86,693,958	0.105	**3.34 × 10^-5^**	ARHGAP24
CB-PWV	7	rs3001450	9	93,164,925	0.61	**3.91 × 10^-5^**	WNK2
CB-PWV	7	rs1389608	14	46,027,527	0.111	**4.08 × 10^-5^**	
RW	7	rs10499221	6	141,000,000	0.003	**5.92 × 10^-5^**	

**2c. Linkage Results**							
	
**Phenotype**	**Exam**	**LOD**	**Chr.**	**Position**	Lower bound*	**Upper bound**	
	
DBP	1–7	2.03	17	12,245,760	9,173,838	16,450,642	
	
SBP	1–7	3	15	100,152,332	97,636,843	100,152,332	
SBP	7	2.55	15	79,161,506	75,509,164	85,958,968	
SBP	7	2.39	3	129,657,137	105,768,506	141,888,352	
SBP	1–7	2.18	5	41,710,612	36,665,015	67,696,396	
SBP	1–7	2.07	3	107,844,505	99,203,989	144,119,612	
SBP	7	2.06	12	101,785,625	94,922,502	107,253,596	
	
RW	7	5.02	8	19,102,897	17,257,073	21,506,898	
RW	7	3.35	9	10,499,434	6,759,229	10,671,522	
RW	7	3.17	4	169,091,021	162,723,480	170,955,956	
CF-PWV	7	3.04	2	74,021,676	49,795,460	103,043,940	
CF-PWV	7	2.68	18	40,229,747	38,788,852	43,206,229	
FW	7	2.47	3	60,298,724	24,621,158	62,757,508	
RW	7	2.47	15	100,152,332	94,749,239	100,152,332	
CF-PWV	7	2.43	15	99,551,603	92,469,518	100,152,332	
RW	7	2.29	1	12,153,078	4,266,833	17,528,974	
CF-PWV	7	2.17	4	11,998,283	7,901,357	25,777,055	
	
**2d. Heritability of Blood Pressure and Arterial Stiffness Phenotypes**							
				
**Phenotype**	**Exam**	**Heritability**	**s.e.**				
				
DBP	1	0.3	0.04				
DBP	7	0.35	0.06				
DBP	1–7	0.55	0.05				
				
SBP	1	0.28	0.04				
SBP	7	0.45	0.06				
SBP	1–7	0.57	0.04				
				
CB-PWV	7	0.02	0.09				
CF-PWV	7	0.43	0.1				
FW	7	0.22	0.09				
MAP	7	0.32	0.1				
RW	7	0.66	0.1				

Association results based on minor allele frequency >= 0.1, HWE p value >= 0.001, call rate >= 0.8

CB-PWV = carotid-brachial pulse wave velocity; CF-PWV = carotid-femoral pulse wave velocity; DBP = diastolic blood pressure; FW = forward wave amplitude; MAP = mean arterial pressure; RW = reflected wave amplitude; SBP = systolic blood pressure

*Lower and upper bounds for LOD-1.5 interval.

FBAT models for an additive genetic effect are presented in Table 2b. Two SNPs for DBP and one for SBP yielded p values < 10^-5^. Of note, rs10520569 in *ADAMTSL3 *was associated with DBP (4.2 × 10^-5^) and SBP (1.4 × 10^-4^). For arterial stiffness phenotypes there were 2 p values < 10^-5^, including rs792833 in *COL8A1*.

Linkage analyses (Table 2c) yielded a LOD score of 3 for long-term SBP on chromosome 15 at 100 Mb. Several tonometry linkage peaks exceeded a LOD score of 3, including a LOD of 5.0 for reflected wave (chromosome 8 at 19 Mb). Heritability estimates (Table 2d) were high for long-term average DBP (h^2 ^= 0.55) and SBP (h^2 ^= 0.57), and intermediate for the other BP phenotypes (h^2 ^= 0.28–0.45). Among the arterial stiffness phenotypes, heritability was high for the reflected arterial waveform (h^2 ^= 0.66), low for carotid-brachial PWV (h^2 ^= 0.02), and intermediate for the other phenotypes (h^2 ^= 0.22–0.43). These heritability results are consistent with our prior findings [6,7].

Secondary analyses using the GEE general genetic effects model (2 degrees of freedom; more sensitive in detecting recessive effects) are presented in Table 3. The lowest p value for long-term DBP was in *CCL20 *(rs7591163, p = 2.3 × 10^-7^) and for SBP was in *CDH13 *(rs3096277, p = 9.9 × 10^-8^). Of note, SNPs in *CDH13*, CCL20, and *WDR69 *were associated with DBP and SBP. GEE general effects models for the tonometry phenotypes identified association of mean arterial pressure with *TGFBR2 *(rs3773643, p = 2 × 10^-7^).

**Table 3:** Results of GEE General Genetic Effects Model for Long-term Average Blood Pressure Phenotypes and Arterial Stiffness

Phenotype	SNP	Chr.	Position	P value*	Gene
**Diastolic Blood Pressure (long-term average)**					

DBP	rs7591163	2	228,423,620	2.90 × 10^-7^	CCL20, WDR69
DBP	rs1901167	5	40,996,921	6.40 × 10^-5^	C7
DBP	rs6829806	4	85,916,019	8.10 × 10^-5^	CDS1
DBP	rs6796000	3	189,874,213	1.10 × 10^-4^	LPP
DBP	rs3096277	16	82,321,705	1.40 × 10^-4^	CDH13
DBP	rs969049	4	99,346,035	1.40 × 10^-4^	
DBP	rs10503497	8	14,326,753	1.40 × 10^-4^	SGCZ
DBP	rs2262138	19	16,213,403	2.10 × 10^-4^	FAM32A, AP1M1
DBP	rs10509333	10	72,737,658	3.70 × 10^-4^	UNC5B, SLC29A3
DBP	rs933296	12	109,837,230	4.10 × 10^-4^	MYL2

**Systolic Blood Pressure (long-term average)**					

SBP	rs3096277	16	82,321,705	9.90 × 10^-8^	CDH13
SBP	rs1721359	2	228,460,118	1.00 × 10^-5^	CCL20, WDR69
SBP	rs225942	14	29,595,139	5.30 × 10^-5^	PRKD1
SBP	rs298988	4	119,867,850	7.80 × 10^-5^	SEC24D
SBP	rs10514096	5	76,700,940	1.10 × 10^-4^	PDE8B
SBP	rs10512245	9	95,771,366	1.40 × 10^-4^	
SBP	rs294593	5	163,000,000	1.80 × 10^-4^	MAT2B
SBP	rs6085660	20	6,639,069	1.90 × 10^-4^	BMP2
SBP	rs6796000	3	190,000,000	2.20 × 10^-4^	LPP
SBP	rs575121	12	117,000,000	2.20 × 10^-4^	TAOK3

**Tonometry**					

MAP	rs3773643	3	30,685,247	1.99 × 10^-7^	TGFBR2
FW	rs3793427	8	17,188,201	1.96 × 10^-6^	VPS37A
RW	rs6492654	13	92,688,671	2.28 × 10^-6^	GPC6
CF-PWV	rs1367248	2	124,734,834	2.88 × 10^-6^	CNTNAP5
CF-PWV	rs10521232	17	13,480,529	3.88 × 10^-6^	HS3ST3A1
FW	rs3766680	1	173,563,0070	4.15 × 10^-6^	TNR
RW	rs1371924	3	144,732,760	4.44 × 10^-6^	SLC9A9
RW	rs10488172	7	132,985,716	8.49 × 10^-6^	EXOC4
FW	rs10507534	13	44,724,220	1.05 × 10^-5^	GTF2F2
FW	rs719856	6	47,702,681	1.21 × 10^-5^	CD2AP

*P values from 2 degree of freedom test

CB-PWV = carotid-brachial pulse wave velocity; CF-PWV = carotid-femoral pulse wave velocity; DBP = diastolic blood pressure; FW = forward wave amplitude; MAP = mean arterial pressure; RW = reflected wave amplitude; SBP = systolic blood pressure

Minor allele frequency >= 0.20, HWE P value >= 0.05, call rate >= 0.80

Geometric means of GEE association results (additive genetic effect model) for SBP and DBP considered jointly are summarized in Table 4. The lowest p values were noted for Examination 1 BP values (rs10493340, p = 1.5 × 10^-5^). Geometric means of association results for the 5 tonometry phenotypes considered concurrently yielded its lowest p value (rs10518082, p = 0.002) for *DCK*.

**Table 4:** Top Results for Geometric Means of SBP and DBP Considered Jointly (at examinations 1, 7 and in the long term), and arterial stiffness phenotypes considered jointly.

SNP	Exam	Chr.	Position	P value	Gene
**DBP and SBP**					

rs10493340	1	1	63,303,150	1.49 × 10^-5^	
rs9321764	7	6	140,532,157	2.89 × 10^-5^	
rs10491334	7	5	110,800,303	3.74 × 10^-5^	CAMK4
rs2121070	1	14	75,720,517	3.96 × 10^-5^	C14orf118
rs1328925	1	4	159,547,895	4.22 × 10^-5^	TMEM144
rs10510079	1	10	122,473,101	7.27 × 10^-5^	
rs7562854	7	2	12,149,816	7.52 × 10^-5^	
rs1841055	7	4	70,039,785	7.63 × 10^-5^	UGT2A3
rs10485320	7	6	47,884,860	7.75 × 10^-5^	OPN5
rs9298203	7	8	73,270,276	8.28 × 10^-5^	

**Arterial stiffness**					

rs10518082	7	4	72,282,885	0.002	DCK
rs1322512	7	6	153,040,067	0.005	SYNE1
rs10511389	7	3	120,557,547	0.007	CDGAP
rs883524	7	8	23,250,536	0.008	LOXL2
rs965674	7	5	82,518,340	0.008	XRCC4
rs10502173	7	11	112,708,233	0.009	TTC12
rs1468512	7	17	64,731,363	0.010	ABCA10
rs4075701	7	2	116,146,020	0.011	
rs10496604	7	2	123,501,987	0.011	
rs770189	7	5	88,124,195	0.011	MEF2C

Based on Additive genetic effects model using GEE and minor allele frequency of 0.1, call rate >= 0.80 and HWE p value >= 0.001

SNPs in 6 renin-angiotensin-aldosterone pathway genes were analyzed for association with the BP and tonometry phenotypes (Table 5). A total of 69 SNPs qualified for analysis (minor allele frequency >= 0.1, Hardy Weinberg equilibrium p >= 0.001, call rate >= 0.8). For the primary traits there were few associations from GEE models for an additive genetic effect with p values < 0.05 and none with p < 0.001.

**Table 5:** Results for Pre-Specified Candidate Genes

Candidate gene	Total number of SNPs*	SNPs with p value < 0.05	Phenotype	GEE p value	FBAT p value
**Diastolic blood pressure**					

ACE	3	0			
AGT	13	rs2478518		0.021	0.186
AGTR1	17	0			
CYP11B2	1	0			
NR3C2	26	rs6845733		0.008	0.399
REN	9	0			

**Systolic blood pressure**					

ACE	3	0			
AGT	13	rs2478518		0.003	0.275
AGTR1	17	0			
CYP11B2	1	0			
NR3C2	26	rs6845733		0.010	0.323
		rs3916013		0.024	0.598
REN	9	0			

**Arterial stiffness**					

ACE	3	0			
AGT	13	rs731824	MAP	0.022	0.397
		rs2478518	FW	0.024	0.688
		rs2478516	FW	0.037	0.217
		rs2478516	RW	0.046	0.663
AGTR1	17	rs1059502	MAP	0.025	0.023
		rs427832	FW	0.046	0.963
CYP11B2	1	rs2717594	CF-PWV	0.003	0.308
NR3C2	26	rs3910046	CF-PWV	0.009	0.383
		rs9307847	CB-PWV	0.011	0.741
		rs3910046	CB-PWV	0.014	0.409
		rs10519959	CB-PWV	0.018	0.175
		rs3846317	RW	0.021	0.649
		rs4835136	CB-PWV	0.027	0.268
		rs3846318	RW	0.042	0.293
		rs10519958	RW	0.049	0.529
REN	9	rs16776	FW	0.012	0.115
		rs3911890	FW	0.022	0.207

*Includes all SNPs within 200 kb of start to 200 kb beyond end of gene, with genotype call rate >= 0.8; minor allele frequency >= 0.1; HWE p >= 0.001

CB-PWV = carotid-brachial pulse wave velocity; CF-PWV = carotid-femoral pulse wave velocity; DBP = diastolic blood pressure; FW = forward wave amplitude; MAP = mean arterial pressure; RW = reflected wave amplitude; SBP = systolic blood pressure

## Discussion and conclusion

We provide results of genome-wide association study for 6 blood pressure and 5 arterial stiffness phenotypes in a carefully characterized study sample. Association analyses and linkage reveal a number of intriguing results. For the GEE model of additive genetic effects (Table 2a) there were 7 SNPs with p values < 10^-5 ^for blood pressure and 5 for arterial stiffness phenotypes. Among the GEE additive effect model results the most likely candidate genes were MEF2C, SYNE1, and TNFSF11, which were associated with arterial stiffness. We have not yet attempted replication of our results. Follow-up genotyping of the top SBP and DBP SNPs reported in our study sample in additional Framingham participants is planned; additional replication attempts will be needed in independent samples to confirm any of the association results we report.

FBAT (Table 2b) identified association of *COL8A1 *with arterial stiffness (p value 6 × 10^-6 ^for rs792833). This gene codes for type VIII collagen, which is produced by aortic endothelial cells [17], suggesting a biologically plausible association.

Linkage yielded a LOD score of 3, approaching genome-wide significance, for long-term SBP on chromosome 15. A meta-analysis of blood pressure and hypertension linkage studies did not identify this as a region of interest [9]. The lower LOD scores for long-term SBP on chromosome 17 (~67 cM) in this investigation compared with our prior findings [6] appears to be largely due to differences in phenotype definition of long-term SBP with the exclusion of early examination BP values in the original cohort participants and the inclusion of offspring cohort examination 7 blood pressures in this analysis. When linkage analyses were repeated with the inclusion of the early original cohort exams using the prior phenotype definitions, the same linkage peak on chromosome 17 emerged (LOD > 4).

For tonometry phenotypes, we found LOD scores for reflected wave amplitude of 5.0 (chromosome 8 at 19 Mb) and 3.2 (chromosome 4, 169 Mb) near peaks for this phenotype that we previously reported in a largely overlapping study sample [7]. Similarly, we once again identified a linkage peak for carotid-femoral pulse wave velocity (LOD 3.0; chromosome 2 at 74 mb).

Compared with the primary GEE model for additive genetic effects (Table 2a), a different set of SNPs was identified in secondary GEE general effects models (Table 3) for long-term DBP and SBP, including 2 SNPs with p values < 10^-6^. Differences in model results may be due to the greater sensitivity of the general model to detect recessive genotype effects. SNPs in *CCL20*, *CDH13*, and *LPP *were associated with both long-term SBP and DBP. GEE general genetic effects models for arterial stiffness phenotypes yielded the lowest p value (p = 1.99 × 10^-7^) for rs3773643 in *TGFBR2*, which has been implicated in aortic aneurysm and Loeys-Dietz syndrome [18,19]. Disruption of the aortic wall would be expected to affect arterial stiffness.

Due to high correlations of SBP and DBP (within examination r = 0.77; long-term r = 0.82), joint analyses of SBP and DBP added little to what was identified in individual phenotype analyses. In contrast, joint analyses of the five tonometry phenotypes, which are less highly correlated, identified *LOXL2*, *SYNE1*, and *MEF2C *as attractive candidates. *LOXL2 *is a member of the lysyl oxidase family of enzymes that initiate cross-linking of collagens and elastin, and alter arterial elasticity [20]. Collagen and elastin cross-links are critical to tensile strength of the extracellular matrix. Mice null for lysyl oxidase (*LOX*) die perinatally from aortic aneurysm [21]. *MEF2C *is involved in cardiac morphogenesis and extracellular matrix remodeling [22]. *SYNE1 *is involved in aortic vascular smooth muscle differentiation [23]. To our knowledge, genetic variation in these genes has not previously been shown to be associated with alterations in arterial properties in humans. Whether our results provide nominal evidence of such association or merely chance findings remains to be determined.

Since none of the primary associations attained genome-wide significance, this investigation should be viewed as hypothesis generating. Association analyses for SNPs in six renin-angiotensin-aldosterone pathway genes showed weak evidence of association. Negative results for these candidate genes may be due in part to incomplete linkage disequilibrium coverage of these genes by the SNPs in this genome-wide scan. It is likely that the vast majority of low p values from association analyses are due to chance. Replication studies in other populations, using a genome-wide approach or selective genotyping is needed to establish if any of our results are indicative of true positive associations.

We provide results of genome-wide association testing for blood pressure and arterial stiffness phenotypes obtained in a carefully described community-based sample of adults who were recruited without regard to disease status. Additional studies are needed to validate these results. Finding genetic variants associated with hypertension or altered arterial properties may aid in the identification of high risk individuals and in the development of new targeted therapies for hypertension. Our report is one of the earlier genome-wide association studies of blood pressure. Several additional studies, some with larger sample size and others with more dense genome-wide coverage of common variation will follow. In that regard, a 550 k SNP genome-wide association study in approximately 9400 Framingham Heart Study participants across three generations is underway and results from that study will help in the interpretation of the findings we report in this manuscript.

## Abbreviations

DBP = diastolic blood pressure; FBAT = family based association test; GEE = generalized estimating equation; LOD = log of the odds; SBP = systolic blood pressure; SNP = single nucleotide polymorphism.

## Competing interests

GFM is owner of Cardiovascular Engineering, Inc, a company that designs and manufactures devices that measure vascular stiffness. All other authors declare that they have no competing interests.

## Authors' contributions

EJB secured funding for tonometry measurements, assisted in planning the analyses, and critically revised the manuscript. CNC contributed to design, analysis, and critical review of the manuscript. SJH generated the phenotype data, participated in the analysis and interpretation of results. MGL assisted to secure funding for tonometry measurements, generated phenotype data, assisted in planning analyses, and critically revised the manuscript. DL conceived of the FHS tonometry project and assisted in securing funding, planned the analyses, interpreted the results, and drafted the manuscript. GFM conceived of the FHS tonometry project and assisted in securing funding, planned the analyses, and critically revised the manuscript. RSV provided critical input in conceiving the project, securing the funding, planning the analyses and critically revising the manuscript. TJW contributed to design, analysis, and critical review of the manuscript.
